# Cytotoxic Potential and Molecular Pathway Analysis of Silver Nanoparticles in Human Colon Cancer Cells HCT116

**DOI:** 10.3390/ijms19082269

**Published:** 2018-08-02

**Authors:** Sangiliyandi Gurunathan, Muhammad Qasim, Chanhyeok Park, Hyunjin Yoo, Jin-Hoi Kim, Kwonho Hong

**Affiliations:** Department of Stem Cell and Regenerative Biotechnology and Humanized Pig Center (SRC), Konkuk Institute of Technology, Konkuk University, Seoul 05029, Korea; gsangiliyandi@yahoo.com (S.G.); qasimattock@gmail.com (M.Q.); chanhyeok.park3751@gmail.com (C.P.); hyunjinyoo7@gmail.com (H.Y.); jhkim541@konkuk.ac.kr (J.-H.K.)

**Keywords:** silver nanoparticles, cell viability, oxidative stress, mitochondria, DNA damage, KEGG analysis, biological pathways

## Abstract

Silver nanoparticles (AgNPs) have gained attention for use in cancer therapy. In this study, AgNPs were biosynthesized using naringenin. We investigated the anti-colon cancer activities of biogenic AgNPs through transcriptome analysis using RNA sequencing, and the mechanisms of AgNPs in regulating colon cancer cell growth. The synthesized AgNPs were characterized using UV–visible spectroscopy (UV–vis), X-ray diffraction (XRD), Fourier-transform infrared spectroscopy (FTIR), dynamic light scattering (DLS), and transmission electron microscopy (TEM). The AgNPs were spherical with sizes of 2–10 nm. Cytotoxicity assays indicated that the AgNPs in HCT116 colorectal cancer cells were very effective at low concentrations. The viability and proliferation of colon cancer cells treated with 5 µg/mL biogenic AgNPs were reduced by 50%. Increased lactate dehydrogenase leakage (LDH), reactive oxygen species (ROS) generation, malondialdehyde (MDA), and decreased dead-cell protease activity and ATP generation were observed. This impaired mitochondrial function and DNA damage led to cell death. The AgNPs upregulated and downregulated the most highly ranked biological processes of oxidation–reduction and cell-cycle regulation, respectively. Kyoto Encyclopedia of Genes and Genomes (KEGG) analysis showed that AgNPs upregulated *GADD45G* in the p53 pathway. Thus, the AgNP tumor suppressive effects were mediated by cell apoptosis following DNA damage, as well as by mitochondrial dysfunction and cell-cycle arrest following aberrant regulation of p53 effector proteins. It is of interest to mention that, to the best of our knowledge, this study is the first report demonstrating cellular responses and molecular pathways analysis of AgNPs in HCT116 colorectal cancer cells.

## 1. Introduction

According to the International Agency for Research on Cancer, colorectal cancer (CRC) is the third most commonly diagnosed malignancy in men and the second most commonly occurring cancer in women [1]. CRC is the third most common cancer and the fourth leading cause of cancer-related deaths worldwide, with nearly 1.4 million new cases diagnosed in 2012. It was predicted that the 2.4 million cases of CRC will increase to 1.36 million for men and 1.08 million for women by 2035 [2]. Thus, it is necessary to reduce the burden of CRC [1,3]. Treatment of CRC remains a major challenge for researchers, gastroenterologists, and oncologists. The compound, 5-fluorouracil, is the only approved drug for treating advanced CRC [4]. Although chemotherapy remains the standard treatment, it causes several side effects such as nausea, vomiting, loss of appetite, constipation or diarrhea, and alopecia. Therefore, alternative therapeutic strategies are needed [5].

Recently, silver nanoparticles (AgNPs) showed potential for therapeutic applications because of their unique properties, and they were used as strong broad-spectrum antimicrobial agents in textiles, food storage containers, antiseptic sprays, catheters, bandages, and anti-cancer agents [6,7]. AgNPs are promising anti-cancer agents. A low concentration of AgNPs causes DNA damage and chromosomal aberrations without significant toxicity [8,9]. Lima et al. observed no genotoxicity effects for different human culture cells treated with up to 10 mg/mL of capped AgNPs with average sizes of 6–80 nm. AgNPs are known to interact with cells and regulate various cellular responses in both passive and active manners [10]. Gurunathan et al. demonstrated the anti-angiogenic properties of AgNPs in vascular endothelial cells, which showed equivalent potency to a natural anti-angiogenic molecule, known as pigment epithelium-derived factor, by inhibiting the phosphorylation of protein kinase B/Akt [11]. SriRam et al. demonstrated the potential cytotoxicity against Dalton’s lymphoma ascites (DLA) and DLA-induced tumors in mice, with significantly increased survival times in the tumor mouse model compared to the untreated group [12]. Low concentrations of poly (*N*-vinyl-2-pyrrolidone)-coated AgNPs inhibited the viability of acute myeloid leukemia cells and the viability of K562 cells in a dose-dependent manner after entering the cells via endocytic pathway endosomes [13]. Importantly, AgNPs were shown to exert anti-cancer effects and exhibited cytotoxicity via different mechanisms on cancer cells, such as by inducing cellular apoptosis through mitochondria-dependent and mitochondria-independent pathways against various types of cancer cells, including human breast cancer cells (MCF-7, MDA-MB-231), lung cancer cells (A549), liver (HepG2), and skin and oral carcinoma cell lines (HT144), via the leakage of lactate dehydrogenase, reactive oxygen species (ROS) generation, and the impairment of mitochondrial dysfunction [14,15,16,17,18,19]. However, the anti-cancer activity of AgNPs depends on various factors such as their size, shape, and surface coatings, as well as the surface charge, the type of cells, and the type of reducing agents used to synthesize the AgNPs. Although conventional physical and chemical methods are simple, the processes are energy-intensive and require toxic chemicals [15]. Additionally, synthetic capping agents such as polyethylene glycol, polyvinyl pyrrolidone, and polyvinyl alcohol are toxic and non-biodegradable. Therefore, the interest in using biomolecules as capping agents for AgNPs increased [20].

The transcriptomics field advanced rapidly in recent years with the introduction of next-generation sequencing technologies, such as RNA sequencing (RNA-Seq), which may displace complementary DNA (cDNA) microarrays as the favored method for gene-expression profiling of cells and tissues [21,22]. Transcriptome analysis associated with bioinformatics data-mining tools can be used to simultaneously analyze numerous genes/targets and to identify the mechanisms of action after treatments. RNA-Seq serves as a useful tool for identifying differentially expressed genes following treatment with various compounds [23]. RNA-Seq has several advantages compared to whole-genome sequencing; it is mainly focused on transcribed regions of genomes, it is free from probe-specific hybridization of microarrays, and it has expansive coverage, allowing for unbiased detection of both coding and noncoding novel transcripts, as well as low-abundance transcripts [23,24,25].

To overcome the limitations of conventional methods, the development of AgNPs with well-controlled morphological and physicochemical features for physiological application in humans is necessary; therefore, we adopted a biological method for synthesizing AgNPs using a pure aqueous solution of naringenin (NAR). Few studies examined the synthesis of AgNPs through pure flavonoid reduction of silver. In this study, we systematically analyzed the anti-cancer potential of the NAR stabilized AgNPs using HCT116 human colon cancer cell lines, and the mechanism of action of AgNPs in regulating the growth of CRC cells using the RNA-Seq approach. Although AgNPs were shown to inhibit cell viability and proliferation in different types of cancer cells, the mechanism is unknown. Therefore, profiling of gene expression can also be used as a new tool for evaluating interactions between nanoparticles and biological systems to reveal molecular mechanisms. Moreover, our method is useful for predicting mechanisms of toxicity of AgNPs in cancer cells.

## 2. Results and Discussion

### 2.1. Synthesis and Characterization of AgNPs Using NAR

**Figure 1:**
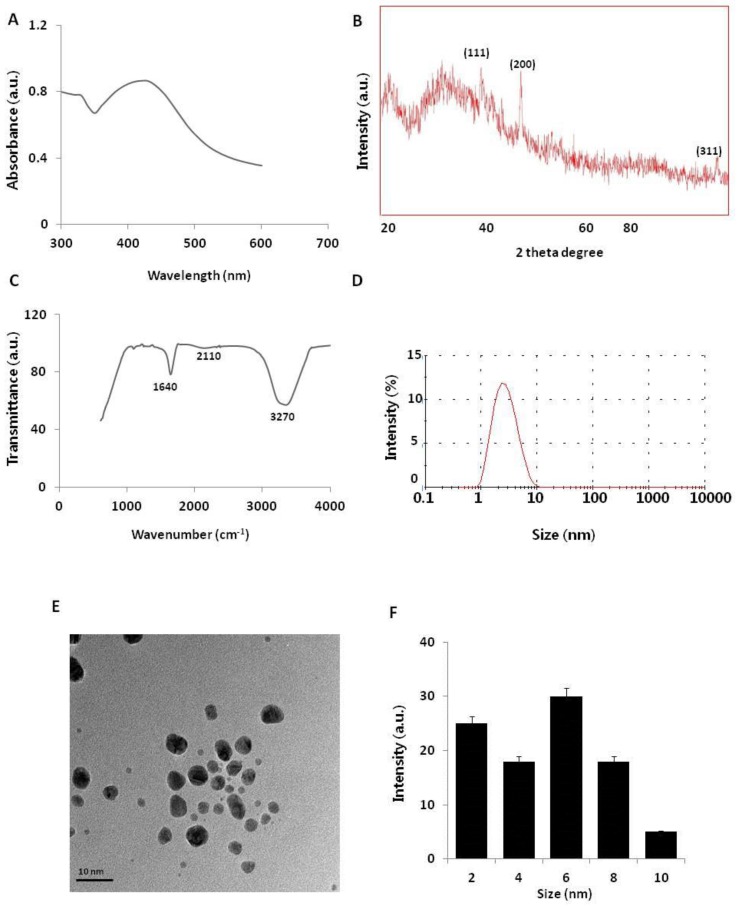
Synthesis and characterization of silver nanoparticles (AgNPs). (**A**) Ultraviolet–visible (UV–vis) spectra of AgNPs. (**B**) X-ray diffraction (XRD) pattern of AgNPs. (**C**) Fourier-transform infrared (FTIR) spectra of AgNPs. (**D**) Size distribution analysis of AgNPs using dynamic light scattering (DLS). (**E**) TEM images of AgNPs. (**F**) Particle size distributions from TEM images. The diameter of AgNPs was determined from more than 200 particles.

The synthesis of AgNPs was carried out using a pure aqueous solution of NAR (50 µM) mixed with 2 mM silver nitrate at 37 °C for 1 h. NAR is a flavonoid with significant antibiotic, anti-cancerous, and anti-inflammatory properties against various types of cancer cells [26,27,28]. Color formation rapidly occurred in this mixture, possibly because of the reaction between Ag ions and NAR [12,28]. The synthesis of AgNPs was confirmed using UV–visible spectroscopy (UV–vis). The UV–visible spectrum showed a single absorption peak at 440 nm, suggesting that the synthesized AgNPs were pure (Figure 1A). In aqueous solution, AgNPs exhibit strong surface plasmon resonance, and their absorbance spectra depend on their size, shape, and morphology. The synthesis of AgNPs using pure flavonoids is faster than using the whole plant extract, and prevents the inclusion of impurities [28,29,30].

To determine the crystalline nature and surface morphology of the synthesized AgNPs, X-ray diffraction (XRD) analysis was performed between the 2θ range of 20° and 80°. Figure 1B shows the XRD pattern of AgNPs synthesized by reacting aqueous silver salt with NAR. The XRD pattern showed characteristic peaks of AgNPs, confirming the crystalline nature of the AgNPs. High-intensity peaks of AgNPs synthesized using NAR were observed at approximately 38°, 44°, and 76°, corresponding to (111), (200), and (311) Bragg reflections, respectively, and are the precise peak positions for the face-centered cubic lattice structure of silver. The average particle size obtained based on the XRD pattern using the Scherrer equation was approximately 6 nm for AgNPs synthesized using NAR. Our findings agree with those of previous reports of AgNPs synthesis using plant extracts [14], using various flavonoids such as hesperidin, NAR, and diosmin [28], and using leaf extracts of *Ocimum Sanctum* and its derivative, quercetin [30].

To determine whether NAR was responsible for reducing silver ions to AgNPs, we performed Fourier-transform infrared (FTIR) spectroscopy analysis. As shown in Figure 1C, the synthesized AgNPs showed peaks at approximately 1640, 2110, and 3270 cm^−1^, which correspond to the groups C=C, C≡C, and amine N–H/O–H stretching vibrations, respectively. This indicates that NAR was responsible for reducing silver ions to AgNPs, which strongly corresponds to the same functional groups present in quercetin responsible for reducing silver into AgNPs [30,31]. Furthermore, IR spectra depicted a strong stretching of the O–H bond as a strong signal peak between 3000 and 3500 cm^−1^ [14,15,17]. A previous study detected a strong signal for an O–H bond in flavonoids used as reducing agents to synthesize AgNPs [32].

Although the size and morphology of the particles can be measured using transmission electron microscopy (TEM), it is important to determine the particle size in solution before examining toxicity in cells. Dynamic light scattering (DLS) methods are used to measure large numbers of particles in a single solution [15,17]. The particle size distribution determined using DLS for the AgNP mixture is shown in Figure 1D. The particle-size histogram indicated that AgNPs varied in size from 1 to 10 nm with a mean diameter of 6 nm. DLS intensity analysis revealed one broad and sharp peak with an average size of 6 ± 1 nm. To determine the uniformity of particle sizes and morphologies, we performed TEM. The TEM image in Figure 1E shows the spherical shape and homogeneous particle size distribution of AgNPs in the micrograph, with sizes close to those determined using DLS. Figure 1F summarizes the size measurement results of AgNPs from the TEM images. Collectively, both TEM and DLS analyses showed that the synthesized AgNPs were 6 nm. Sahu et al. reported that the sizes of nanoparticles synthesized from hesperidin, diosmin, and NAR were approximately 5–50 nm, 5–40 nm, and 20–80 nm, respectively [28]. Hesperidin- and NAR-derived AgNPs were oval-shaped and polydispersed, while diosmin-derived AgNPs were hexagonal-shaped. Prathna et al. produced AgNPs with an average size of 50 nm using citrus plant extract [33]. Jain and Mehata reported that Tulsi extract- and quercetin-mediated synthesis of AgNPs had average sizes of 14.6 and 11.35 nm, respectively [30]. Our findings suggest that NAR produced even smaller particles, which can easily penetrate cells and release silver ions faster compared to larger particles.

### 2.2. Effect of AgNPs on Cell Viability and Proliferation of HCT116 and HT-29 Cells

**Figure 2:**
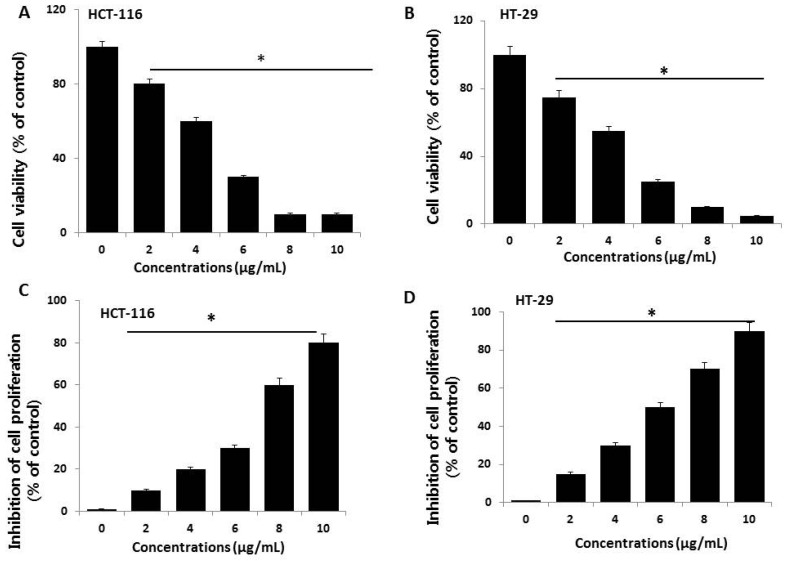
Cell viability and proliferation assessment of AgNPs in HCT116 and HT-29 cells. (**A**,**B**) Viability of HCT116 and HT-29 cells was determined 24 h after exposure to different concentrations of AgNPs with a Cell Counting Kit-8 (CCK-8) assay. The results are expressed as the mean ± standard deviation of three independent experiments. A significant difference was observed from 2 µg/mL onward. (**C**,**D**) Cell proliferation of HCT116 and HT-29 was performed using a BrdU cell proliferation assay. The results are expressed as the mean ± standard deviation of three independent experiments. A significant difference was observed from 2 µg/mL onward. There was a significant difference in the ratio for AgNP-treated cells compared to untreated cells, determined by a Student’s *t*-test (* *p* < 0.05).

To evaluate the potential toxicity effects of AgNPs, HCT116 and HT-29 cells were treated with various concentrations of AgNPs (2–10 µg/mL), and cell viability was determined based on mitochondrial activity. After 24 h of exposure, mitochondrial activity was decreased in response to a concentration of 2 µg/mL, and the cell viability rapidly decreased when AgNP concentrations were increased from 2 to 10 µg/mL (Figure 2A). At 4–10 µg/mL AgNPs, mitochondrial activity significantly decreased to 50% for cells exposed to 5 and 4 µg/mL AgNPs in HCT116 and HT-29 cells, respectively. At this time point and dose, mitochondrial activity was significantly reduced in AgNP-exposed cells. Miethling-Graff et al. [34] reported the size-dependent (10, 20, 40, 60, and 100 nm) effects of AgNPs in the human LoVo cell line. The authors found that cellular uptake and toxicity were regulated by size, and smaller particles easily penetrated the cells, while larger particles such as 100-nm particles did not. Similarly, *para*-hydroxybenzoate tetrahydrate (SPHT)-assisted synthesis of AgNPs showed dose- and time-dependent inhibition of cell viability in the human colon cancer cell lines, HCT15 and HT-29. Cells exposed to SPHT-AgNPs (8 µg/mL) displayed 50% inhibition of cell proliferation after 24 h of treatment [35]. Plant-assisted AgNPs showed greater anti-proliferative effects against HCT116 cells at a minimum concentration of approximately 450 nM than synthetic AgNPs and AgNO_3_ [36]. *Abutilon indicum*-assisted AgNPs showed dose-dependent anti-proliferative effects against COLO 205 (human colon cancer) and MDCK (normal) cells [37].

Next, we examined the dose-dependent effects of various concentrations of AgNPs on cell proliferation by measuring BrdU incorporation during DNA synthesis. HCT116 cells exposed to various concentrations of AgNPs showed a significantly reduced proliferation rate compared to control cells, which agrees with the results of the cell viability analysis (Figure 2B). The proliferation rate, relative to non-exposed control cells, was evaluated after a 24-h exposure to various concentrations of AgNPs. The results revealed that the proliferation rate decreased with increasing doses. Similarly, AgNPs exhibited anti-proliferative effects in various types of cancer cells including human breast cancer cells [8,38], human lung cancer cells [17], human ovarian cancer cells [15], and human neuroblastoma cells [39]. Among these two tested cell lines, HT-29 seems to be sensitive compared to HCT116 cells. Therefore, further study focused on HCT116 cells.

### 2.3. AgNPs Increase Cytotoxicity in HCT116 Cells

**Figure 3:**
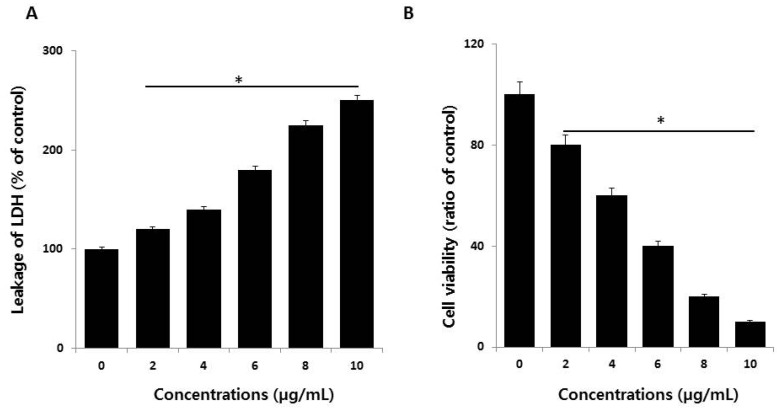
Measurement of lactate dehydrogenase (LDH) leakage and cell-death protease activity. (**A**) LDH activity was measured at 490 nm using an LDH cytotoxicity kit. (**B**) The level of dead-cell protease was determined using the CytoTox-Glo cytotoxicity assay. The results are expressed as the mean ± standard deviation of three independent experiments. There was a significant difference in the ratio for AgNP-treated cells compared to untreated cells, determined by a Student’s *t*-test (* *p* < 0.05).

The lactate dehydrogenase (LDH) leakage assay is a well-known cytotoxicity assay for measuring cytotoxicity based on the leakage of intracellular molecules through impaired plasma membranes. LDH is a soluble cytoplasmic enzyme present in nearly all cells, and it is released into the extracellular space when the plasma membrane is damaged [16,40,41]. To detect the leakage of LDH into cell culture medium, the cells were treated with different concentrations of AgNPs (2–10 µg/mL) for 24 h, and then, leakage was measured as the amount of formazan product formed using standard spectroscopy. As expected, increasing doses of AgNPs showed that leakage of LDH was directly proportional to the dosage of AgNPs and increased cytotoxicity (Figure 3A), indicating that cells undergoing accidental cell death upon exposure to AgNPs swell and lose membrane integrity before shutting down and releasing their intracellular contents into the surrounding environment. These results indicate that high concentrations of AgNPs damage cellular membrane integrity. Our results agree with those of previous reports showing that AgNPs induce leakage of LDH in different type of cancer cells and non-cancer cells, including human breast cancer cells [14,38], human lung cancer cells [17], human ovarian cancer cells [16], human neuroblastoma cells [39], human microvascular endothelial cells [42], male somatic cells, spermatogonial stem cells [8], and neural stem cells [43]. Collectively, the results suggest that reduced viability was observed in HCT116 cells exposed to AgNPs, which correlates with the increased leakage of LDH and significantly strong cytotoxicity.

Although several assays are established for measuring cell viability and toxicity, proteolytic activities associated with cell death or viability are very sensitive and depend on membrane integrity, acting as cytotoxic agents. Therefore, we measured the impact of AgNPs on dead-cell protease activity in HCT116 cells exposed to various concentrations of AgNPs; the cell viability was calculated according to the manufacturer’s instructions (Promega Corp., G9292, WI, USA) and a previously reported method [44]. HCT116 cells treated with AgNPs showed reduced viability with increasing concentrations of AgNPs (Figure 3B). These results suggest that the viability of HCT116 cells was profoundly reduced, which agrees with the results of the cell viability and LDH leakage assays.

### 2.4. Effect of AgNPs on ROS Generation and Malondialdehyde (MDA)

**Figure 4:**
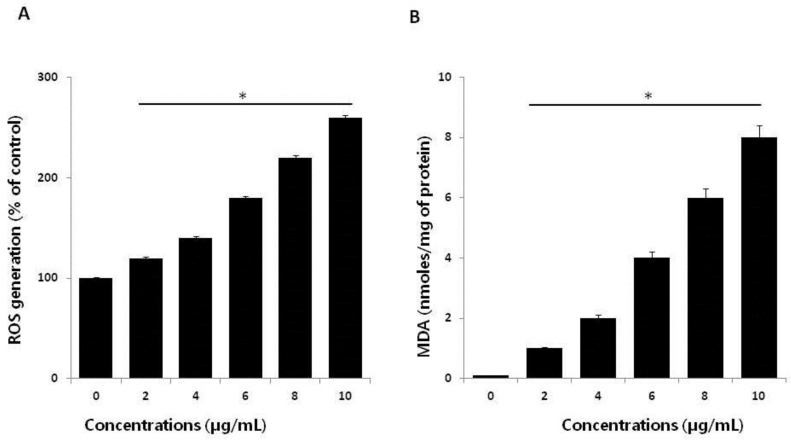
Effect of AgNPs on reactive oxygen species (ROS) generation. (**A**) HCT116 cells were treated with or without AgNPs for 24 h, and ROS generation was measured using 2′,7′-dichlorodihydrofluorescein diacetate (DCFH-DA). (**B**) HCT116 cells were treated with AgNPs for 24 h. After incubation, the cells were harvested and washed twice with ice-cold phosphate-buffered saline (PBS) solution. The cells were collected, and disrupted by ultrasonication for 5 min on ice. Lipid peroxidation (LPO) was determined via the reaction of malondialdehyde (MDA) with thiobarbituric acid to form a colorimetric (532 nm)/fluorometric (excitation and emission wavelengths of 532 and 553 nm, respectively) product, whose quantity was proportional to that of MDA. The results are expressed as the mean ± standard deviation of three independent experiments. There was a significant difference in the ratio for AgNP-treated cells compared to untreated cells, determined by a Student’s *t*-test (* *p* < 0.05).

Ag ions play an important role in catalyzing ROS production in the presence of oxygen species, and AgNPs can induce oxidative stress in a variety of cellular systems by generating ROS, including in human lung cancer A549 cells [45,46], human ovarian cancer cells [16], and human neuroblastoma cells [39]. To examine the effect of AgNPs on ROS generation, HCT116 cells were treated with various concentrations of AgNPs for 24 h, and then, ROS generation was measured in a an H_2_DCF-DA assay. After 24 h of exposure, increasing concentrations of AgNPs (2–10 µg/mL) significantly increased ROS levels at concentrations above 6 µg/mL compared to the control (Figure 4A). ROS levels generated in response to AgNPs treatment were significantly higher than those in normal cells. At all tested concentrations, the most pronounced effects were observed at higher concentrations, showing up to 2–3-fold higher ROS levels. Miethling-Graff et al. [34] reported the size-dependent toxicity by generation of ROS in the human LoVo cell line, in which smaller-sized particles produced more ROS than larger-sized particles. Mata et al. [37] found that COLO205 cells exposed to AgNPs produced significant levels of ROS compared to untreated control cells. The major anti-cancer effect of AgNPs involves the induction of apoptosis and autophagy through various molecular mechanisms, including by significantly decreasing cell viability and motility, impairing matrix metalloproteinase-2 and -9 activity, and promoting ROS generation, all of which induce cell death through apoptosis and autophagy [47]. The potential toxicity of AgNPs depends on ROS generation and depletion of the antioxidant defense systems, as well as the loss of mitochondrial membrane potential [44]. The influence of the surface coating of AgNPs by various biological reducing agents, such as bacterial cellular extracts and fungal cellular extracts, affects ROS generation in human breast cancer cells. For example, fungal extract-mediated synthesis of AgNPs induced more ROS generation than AgNPs synthesized using bacterial cellular extracts, suggesting that coating materials influence ROS generation, and ultimately, cell death [14]. In contrast, chitosan-derived polysaccharide-coated AgNPs showed antimicrobial activity, with no toxicity observed against eukaryotic cells [48]. Collectively, our findings suggest that AgNPs generate significant levels of ROS, which may induce apoptosis by disturbing homeostasis between the oxidant and antioxidant enzyme systems to alter cellular redox [44].

ROS-mediated oxidative stress is a common mechanism of cell death, and it is known to regulate various cellular functions such as cellular proliferation, apoptosis, and necrosis depending on the level of levels of oxidant and antioxidant enzymes [49]. Exposure of cancer cells to chemotherapeutic agents, cytotoxic agents, and nanoparticles creates an imbalance between pro-oxidants and antioxidants. Thus, we analyzed the level of MDA in HCT116 cells treated with various concentrations of AgNPs for 24 h. The results indicated that AgNPs increased the level of MDA; moreover, increasing concentrations of AgNPs significantly increased the MDA levels in human prostate cancer cells (Figure 4B). Gliga et al. [50] reported that long-term exposure of AgNPs in human lung cells upregulated the gene expression of antioxidant enzymes such as glutathione-*S*-transferase enzymes involved in clearing lipid peroxidation products. Lipid peroxidation is a source of free-radical formation and a hallmark of ferroptosis, which is emerging as a form of regulated cell death. Collectively, our findings suggest that AgNPs induce oxidative stress through the oxidation of lipid biomolecules.

### 2.5. AgNPs Induce Mitochondrial Dysfunction and Reduce ATP Generation in HCT116 Cells

**Figure 5:**
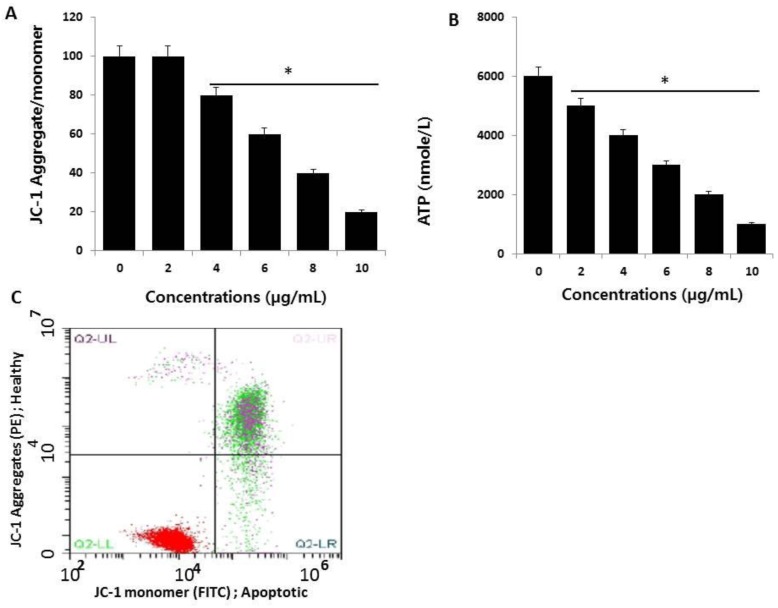
Measurement of mitochondrial membrane potential (MMP) and ATP. (**A**) HCT116 cells were treated with AgNPs for 24 h. The change in MMP was determined using the cationic fluorescent dye, JC-1 (Molecular Probes, Eugene, OR, USA). (**B**) Flow cytometric analysis (FACS) of the mitochondrial membrane potential using the JC-1 dye in HCT116 cells treated with AgNPs (half maximal inhibitory concentration (IC_50_) 3 µg/mL) for 24 h. (**C**) The ATP level was measured according to the manufacturer’s instructions (Sigma-Aldrich, St. Louis, MO, USA; Catalog Number MAK135) in HCT116 cells exposed to AgNPs for 24 h. The results are expressed as the mean ± standard deviation of three independent experiments. There was a significant difference in the ratio of AgNP-treated cells compared to untreated cells, determined by a Student’s *t*-test (* *p* < 0.05).

Loss of mitochondrial function is a major determinant and indicator of cell death, which can be assessed by monitoring changes in mitochondrial membrane potential (MMP). We measured MMP in AgNP-treated HCT116 cells using cationic fluorescent dyes [47]. To determine the influence of AgNPs on MMP, HCT116 cells were treated with various concentrations of AgNPs for 24 h, and then, the MMP status was measured. The treated cells showed remarkable differences compared to control cells (Figure 5A). These results indicate that AgNP incubation for 24 h severely reduced MMP, and that the reductions in cell viability and toxicity were associated with the loss of MMP and increased accumulation of ROS. Previous studies reported that increased ROS generation leads to mitochondria-dependent cell death pathways through the formation of mitochondrial permeability transition pores [51,52]. Mitochondrial membrane depolarization is known to increase following ROS production, which is major factor activating intrinsic cell-death pathways [16,46]. Therefore, the loss of MMP supports mitochondrial depolarization as a primary mechanism of AgNP-induced toxicity, involving one or more signaling cascades with crosstalk between the mitochondrion and other cellular components [45,53,54]. As a result of cellular uptake of AgNPs by HCT116 cells, the cytotoxicity was increased via an alteration of MMP. The change in MMP was further confirmed using flow cytometry analysis of AgNP-treated HCT116 cells using JC-1 dye. The mitochondrial stain, JC-1, can spontaneously aggregate, emitting red fluorescence at high membrane potential in healthy cells, whereas in the apoptotic cells, JC-1 remains in the form of green fluorescent monomers due to mitochondrial membrane depolarization. The change in MMP in HCT116 cells induced by AgNPs is shown in Figure 5B. This indicates that the induction of apoptosis by AgNPs is associated with the mitochondrial pathway.

Next, to confirm whether the loss of MMP influences ATP production in AgNP-treated cells, we measured ATP levels in both AgNP-treated and untreated cells. It is well known that the mitochondria provide most of the energy required for proper cellular function, and damage to the mitochondria results in decreased or inefficient energy production, potentially affecting ATP production or ATP-dependent cellular mechanisms [55]. As expected, AgNP-treated cells showed significantly lower levels of ATP production compared to controls (Figure 5B). Interestingly, increasing concentrations of AgNPs led to decreased ATP production. The results showed that there was a direct linear relationship between AgNP concentration and ATP production. Thus, the cells appear to be sensitive to mitochondrial toxicants such as AgNPs, which is similar to drug-induced mitochondrial poisoning-inherited mitochondrial diseases [56].

### 2.6. AgNPs Induce Apoptosis

**Figure 6:**
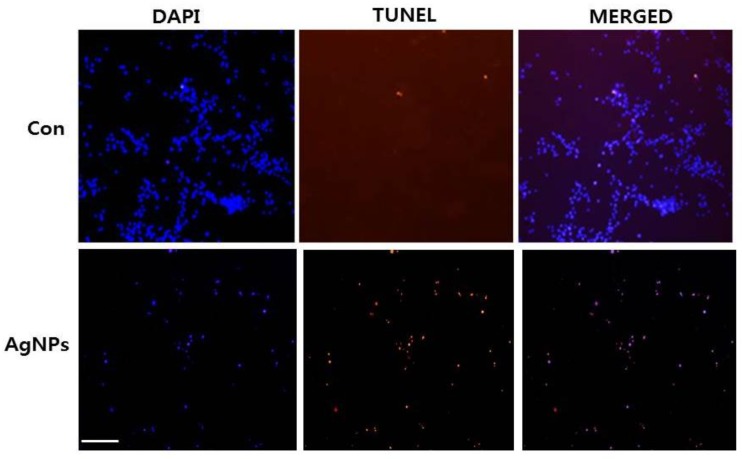
AgNP-induced apoptosis in HCT116 cells. HCT116 cells were treated with AgNPs for 24 h. Next, cell apoptosis was assessed using the terminal deoxynucleotidyl transferase-mediated dUTP nick end labeling (TUNEL) assay; nuclei were counterstained with 4′,6-diamidino-2-phenylindole (DAPI). Representative images show apoptotic (fragmented) DNA (red staining) and the corresponding cell nuclei (blue staining). Original magnification, ×100; scale bar, 100 µm.

High ROS levels induce oxidative stress and cell death [57]. Previously, our lab and other research groups showed that AgNP-induced cytotoxicity primarily results from oxidative stress which, in turn, induces cell death through activation of the intrinsic pathway of apoptosis, autophagy, or both [15,39,46,53]. We examined whether AgNP-induced cell death occurs via apoptosis using terminal deoxynucleotidyl transferase-mediated dUTP nick end labeling (TUNEL) analysis. Oxidative stress is a crucial factor for inducing apoptosis in cancer cells through various mechanisms causing macromolecular damage, such as lipid peroxidation, DNA fragmentation, protein denaturation, and mitochondrial dysfunction [53]. ROS can act as signal molecules to promote cell-cycle progression and induce oxidative DNA damage [51,58]. Measurement of DNA fragmentation appears to be the best technique for evaluating AgNP-induced apoptosis. To detect apoptotic features induced by AgNPs, HCT116 cells were treated with the half maximal inhibitory concentration (IC_50_) of AgNPs, and a DNA-fragmentation assay was conducted. The results, shown in Figure 6, revealed a significant number of positively labeled cells, representing apoptotic DNA fragmentation. In control cultures, few or no apoptotic cells were observed. Characteristic features of apoptosis include cell shrinkage, chromatin condensation, extensive plasma membrane bleb, and separation of cell fragments into apoptotic bodies [59]. Cancer cell lines treated with AgNPs exhibited the same “laddering” pattern as evidenced by DNA fragmentation observed in this study [14]. During DNA fragmentation, the deposition of silver particles inside the nucleus may affect DNA and cell division, and nanoparticles may induce dose-dependent DNA damage, chromosomal aberrations, and errors in chromosome segregation, as well as the formation of sister chromatic exchanges [60]. Our results are consistent with those of previous studies showing that cancer cells treated with AgNPs induce the production of micronuclei [51]. Therefore, we found that AgNPs induce DNA fragmentation, and eventually, induce apoptosis in HCT116 cells.

### 2.7. AgNP Treatment Impairs Expression of Genes Involved in Mitochondrial Function and Cell Apoptosis

**Figure 7:**
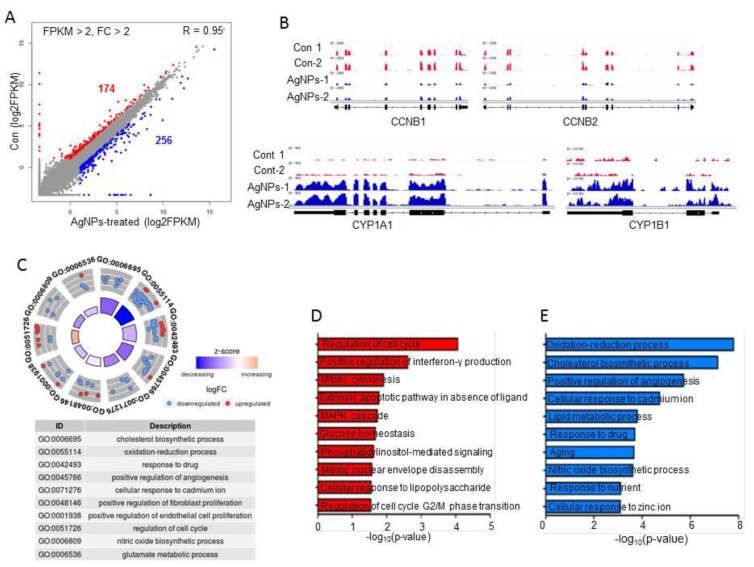
Impact of AgNPs on gene expression. (**A**) Scatter plot showing up- and downregulated genes in AgNP-treated HCT116 cells. The log_2_ fragments per kilobase of transcript per million mapped reads (FPKM) values were used for plotting. Red and blue dots indicate downregulated and upregulated genes in AgNP-treated HCT116 cells, respectively. Cut-off: FPKM ˃2 and fold change ˃2. (**B**) Representative IGV images of down- (*CCNB1* and *CCNB2*) and upregulated (*CYP1A1* and *CYP1B1*) genes. (**C**) Gene Ontology (GO) term analysis using both up- and downregulated genes. Each red (downregulated) or blue (upregulated) dot represents one gene belonging to each term. (**D**,**E**) bar graphs showing GO terms of individual up- and downregulated genes.

To identify which genes were altered by AgNP treatment, cells were treated with the 50% inhibitory concentration of AgNPs (5 µg/mL), and then, we performed RNA-Seq analysis and generated ~26 million reads of RNA-Seq data in each sample. As shown in Figure 7A, 256 upregulated and 174 downregulated genes were identified with cut-off values of fragments per kilobase of transcript per million mapped reads (FPKM) ˃2, and fold changes ˃2 in AgNP-treated HCT116 cells. Representative upregulated (*CYP1A1* and *CYP1B1*) and downregulated genes (*CCNB1* and *CCNB2*) upon AgNP treatment are illustrated in Figure 7B. Both upregulated and downregulated genes were subjected to Gene Ontology (GO) term analysis to identify biological processes. The highest-ranked biological processes included oxidation–reduction processes, responses to drug, cellular responses to cadmium ions, and regulation of the cell cycle (Figure 7C). Next, each up- or downregulated gene was subjected to GO term analysis. The data further support our findings of the cellular and molecular analyses. As shown in Figure 7D,E, the most highly ranked biological process with upregulated genes was oxidation–reduction, whereas the most highly ranked biological process with downregulated genes was regulation of the cell cycle. Particularly, the oxidation–reduction-related genes code for cytochrome P450 monooxygenases such as CYP1A1 and CYP1B1. The cytochrome P450 enzymes are mainly distributed in the endoplasmic reticulum and mitochondria, and they catalyze many reactions involved in steroid and cholesterol synthesis and drug metabolism. A previous study showed that mitochondrial CYP1B1 is involved in melatonin-induced apoptosis in the SH-SY5Y neuroblastoma cell line. It is well known that melatonin has dual functions in apoptosis; anti-tumorigenic effects were observed in some cancer cells by promoting apoptosis, whereas no or little effect was observed in normal cells. Consistent with our data, forced expression of *CYP1A1* in cancer cells led to apoptosis. Asik et al. [61] also showed that administration of a high dose of cobalt ferrite magnetic nanoparticles to human breast cancer cell lines caused apoptosis with elevations in *CYP1A1* and *CYP1B1* expression. Interestingly, genes linked to aging were selectively upregulated in AgNP-treated cells. In contrast, G2/M check-point regulators including *CCNB1* and *CCNB2* were found in the biological process of regulation of the cell cycle with downregulated genes. Multiple bio-grade chemicals were shown to block cell-cycle progression by repressing *CCNB1* or *CCNB2* gene expression, eventually leading to cell apoptosis.

### 2.8. AgNP Treatment Dysregulates Multiple Biological Pathways

**Figure 8:**
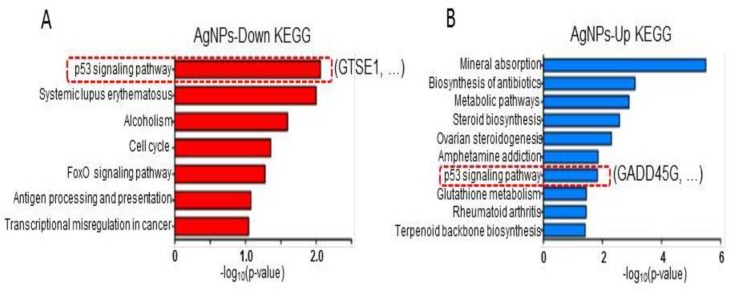
KEGG pathway analysis. (**A**,**B**) Bar graphs showing biological pathways associated with down- and upregulated genes following AgNP administration. The p53 pathway is highlighted with a representative gene in each case.

Next, to examine the biological pathways associated with the differentially expressed genes, KEGG pathway analysis was performed. As shown in Figure 8A,B, p53 signaling and cell-cycle pathways were detected for downregulated genes, whereas mineral absorption and metabolic pathways were detected for upregulated genes. Interestingly, the p53 pathway was found in both up- and downregulated genes. An S/G2 phase-specific gene, *GTSE1*, which is an AgNPs-mediated repressed gene in the p53 pathway, promotes the degradation of p53 by forming a protein complex. Additionally, elevation of *GTSE1* gene expression was detected in multiple cancer types including lung cancer, myeloma cells, and gastric cancer. *GADD45G*, a downstream regulator of p53, was shown to be downregulated in multiple cancer types. Hsu et al. [62,63] showed that induction of *GADD45G* by treatment with a natural compound, cucurbitacin E, caused G2/M arrest in malignant glioma GBM 8401 cells. Our KEGG analysis identified *GADD45G* in the p53 pathway with genes upregulated by AgNPs. Our results agree with those of Gurunathan et al. [15] showing involvement of p53 and the critical role of p53 upregulation in AgNP-mediated cell death in human breast cancer cells. Therefore, our genome-wide study suggests that the tumor-suppressive role of AgNPs is mediated via combinatorial effects of cell apoptosis by DNA damage, and mitochondrial dysfunction and cell cycle arrest by aberrant regulation of p53 effector proteins.

## 3. Materials and Methods

### 3.1. Materials

HCT116 colorectal cell lines were purchased from the Korean Cell Line Bank (Seoul, Korea). Penicillin-streptomycin, trypsin-EDTA, Dulbecco’s modified Eagle’s medium (DMEM), and 1% antibiotic-antimycotic were obtained from Life Technologies/Gibco (Grand Island, NY, USA). Fetal bovine serum and the in vitro toxicology assay kit were purchased from Sigma-Aldrich (St. Louis, MO, USA). Silver nitrate and all other chemicals were purchased from Sigma-Aldrich unless otherwise stated.

### 3.2. Synthesis and Characterization of AgNPs

Synthesis and characterization of AgNPs was carried out as described previously [17]. Synthesis of AgNPs was carried out using naringenin (NAR) dissolved in dimethyl sulfoxide. AgNPs were synthesized by incubating 50 µM NAR in 100 mL of water containing 2 mM AgNO_3_ at 37 °C for 1 h. The color change from pale yellow to dark yellowish-brown was attributed to the formation of AgNPs in the reaction mixture.

### 3.3. Cell Viability and Cell Proliferation

Cell viability was measured using a Cell Counting Kit-8 (CCK-8; CK04-01, Dojindo Laboratories, Kumamoto, Japan). Cell proliferation was determined according to the manufacturer’s instructions (Roche, Basel, Switzerland). Briefly, HCT116 cells were plated in 96-well flat-bottom culture plates containing various concentrations of AgNPs. After 24-h culture at 37 °C and 5% CO_2_ in a humidified incubator, CCK-8 solution (10 μL) was added to each well, and the plate was incubated for another 2  h at 37 °C. Absorbance was measured at 450 nm using a microplate reader (Multiskan FC; Thermo Fisher Scientific, Inc., Waltham, MA, USA).

### 3.4. Membrane Integrity

The membrane integrity of HCT116 cell lines was evaluated using an LDH Cytotoxicity Detection Kit. Briefly, cells were exposed to various concentrations of AgNPs for 24 h. Subsequently, 100 μL of cell-free supernatant from each well was transferred in triplicate into the wells of a 96-well plate, and then, 100 μL of the LDH reaction mixture was added to each well. After 3 h of incubation under standard conditions, the optical density of the final solution was determined at a wavelength of 490 nm using a microplate reader.

### 3.5. Assessment of Dead-Cell Protease Activity

Dead-cell protease activity was assessed as previously described [44]. The cytotoxicity assay was used to evaluate the cytotoxic effects of AgNPs in HCT116 cells. Cytotoxicity was determined using the reaction of intracellular protease with a luminogenic peptide substrate (alanyl-alanylphenylalanyl-aminoluciferin). Luminescence was measured using a Luminescence Counter (Perkin Elmer, Waltham, MA, USA).

### 3.6. Determination of Intracellular ROS

HCT116 cells were treated with AgNPs for 24 h. ROS were measured according to a previous method based on the intracellular peroxide-dependent oxidation of 2′,7′-dichlorodihydrofluorescein diacetate (DCFH-DA; Molecular Probes, Eugene, OR, USA) to form the fluorescent compound 2′,7′-dichlorofluorescein (DCF) [45,46].

### 3.7. Measurement of MDA

Oxidative stress markers, such as malondialdehyde (MDA), were assayed according to each manufacturer’s instructions. Briefly, the cells were cultured in 75-cm^2^ culture flasks and exposed to various concentrations of AgNPs for 24 h. The cells were harvested in chilled phosphate-buffered saline (PBS) by scraping and washing twice with saline, followed by centrifugation at 4 °C for 6 min at 1500 rpm. The cell pellet was sonicated at 15 W for 10 s (three cycles) to obtain the cell lysate. The resulting supernatant was stored at −70 °C until analysis.

### 3.8. JC-1 Assay

HCT116 cells were treated with AgNPs for 24 h. Changes in mitochondrial membrane potential (MMP) were evaluated using the cationic fluorescent dye, JC-1 (Molecular Probes). Fluorescence of JC-1 aggregates and JC-1 monomers was measured at an excitation wavelength of 488 nm and emission wavelengths of 583 and 525 nm using a Gemini EM fluorescence microplate reader (Molecular Devices, Sunnyvale, CA, USA).

### 3.9. Measurement of ATP

The ATP level was measured according to the manufacturer’s instructions (Sigma-Aldrich; Catalog Number MAK135) in HCT116 cells exposed to various concentrations of AgNPs for 24 h.

### 3.10. TUNEL Analysis

To detect apoptotic cells in groups treated with AgNPs (5 µg/mL), the terminal deoxynucleotidyl transferase-mediated dUTP nick end labeling (TUNEL) method was employed using an in situ detection kit (Promega, Madison, WI, USA) according to the manufacturer’s instructions. HCT116 cells were plated in six-well plates (2 × 10^5^ cells per well) and incubated with AgNPs (5 µg/mL) for 24 h, and then, cell apoptosis was quantified using TUNEL analysis. Samples were evaluated under a Nikon Eclipse E400 fluorescence microscope (Nikon 40× Plan 40/0.65, Tokyo, Japan). Differences between the number of TUNEL-positive cells in the control and experimental samples were statistically analyzed.

### 3.11. RNA-Seq and Downstream Bioinformatics Analysis

Total RNA was extracted using TRIzol reagent following the manufacturer’s instructions (Thermo Fisher Scientific, Waltham, MA, USA). The quality of total RNA was evaluated using the Agilent 2100 bioanalyzer (Agilent Technologies, Santa Clara, CA, USA) with the RNA 6000 Nano LabChip kit. RNA-seq libraries were prepared using the Illumina^®^ TruSeq stranded total RNA Library Prep Kit v2 (San Diego, CA, USA), and were sequenced using an Illumina^®^ Hiseq2500 to obtain 150-base pair paired-end reads. The sequencing depth of each sample was >20 million reads. The reads were aligned with TopHat 2.0.13 to GRCh37 with default parameters, and were then assembled by Cufflink 2.2.1 using Ensembl v75 annotations. Transcript abundance was measured in fragments per kb of exon per million fragments mapped (FPKM). Differentially expressed genes were identified at an FPKM ˃2 and fold change ˃2. The RNA-seq data are available on the GEO (GSE100687). The enrichment of biological processes and KEGG pathways was conducted using DAVID (v6.8) tool. The R package (v3.3.2) and GOplot package (version 1.0.2) were used to generate scatter plots and Gene Ontology terms, respectively. Integrative genomics viewer was used to visualize the expression patterns of representative genes.

### 3.12. Statistical Methods

Independent experiments were repeated at least three times, and the data are represented as the mean ± standard deviation for all duplicates within an individual experiment. Data were analyzed using a *t*-test, or multivariate analysis, or one-way analysis of variance, followed by the Tukey test for multiple comparisons, to detect differences between groups, denoted by an asterisk, using the Graph-Pad Prism analysis software (GraphPad, Inc., La Jolla, CA, USA).

## 4. Conclusions

Despite considerable focus on the anti-cancer effects of AgNPs in recent years, studies of the potential effects and molecular mechanism of AgNPs in cancer are limited. Therefore, the aim of this study was to explore the effects of AgNP exposure on human colorectal cancer cells, and to investigate the molecular mechanism using RNA-Seq. The human HCT116 cell line was exposed to 2–10 μg/mL AgNPs (6 nm) for 24 h followed by various cellular assays and RNA-Seq analysis. To determine the potential anti-cancer effect of AgNPs, we synthesized AgNPs using naringenin, which is a flavonoid predominantly found in grapefruit. HCT116 cells exposed to AgNPs for 24 h exhibited significant loss of cell viability and proliferation in a dose-dependent manner. The 50% inhibitory concentrations of 5 µg/mL AgNPs remarkably increased LDH leakage, ROS generation, and MDA levels, and decreased dead-cell protease activity levels and ATP generation. These events led to cell death by impairing mitochondrial function and by inducing DNA damage. Furthermore, our study also demonstrated that AgNPs upregulate and downregulate the most highly ranked biological processes of oxidation–reduction and regulation of the cell cycle, respectively. Our KEGG analysis identified *GADD45G* in the p53 pathway with genes upregulated by AgNPs. Therefore, our results suggest that the tumor-suppressive role of AgNPs is mediated via combinatorial effects of cell apoptosis by DNA damage, and mitochondrial dysfunction and cell cycle arrest by aberrant regulation of p53 effector proteins. Transcriptomic analysis showed that a substantial number of genes, 256 upregulated and 174 downregulated, were differentially expressed following AgNPs exposure; significant effects were observed in genes associated with oxidation–reduction and regulation of the cell cycle. Downstream analysis of the transcriptomics data identified several affected pathways, including the p53 pathway. In conclusion, using a combination of RNA-Seq and functional assays, our study revealed that exposure of human CRC cells to AgNPs induces cell apoptosis via DNA damage, mitochondrial dysfunction, and cell-cycle arrest by aberrant regulation of p53 effector proteins. Moreover, gene-expression profiling methods, such as RNA-Seq, can be used to predict mechanisms of toxicity of AgNPs. GO analyses demonstrated the molecular mechanism of the anti-cancer effect of AgNPs. The present study showed that transcriptome analysis can provide insights into the molecular mechanism of the anti-cancer activity of biogenic AgNPs.
